# Simplified technique of laparoscopic cholecystectomy in a patient with situs inversus: a case report and review of techniques

**DOI:** 10.1186/s12893-015-0012-6

**Published:** 2015-03-11

**Authors:** Natthawut Phothong, Thawatchai Akaraviputh, Vitoon Chinswangwatanakul, Atthaphorn Trakarnsanga

**Affiliations:** Department of Surgery, Panyananthaphikkhu Chonprathan Medical Center, Srinakharinwirot University, Nonthaburi, Thailand; Minimally Invasive Unit, Department of Surgery, Faculty of Medicine Siriraj Hospital, Mahidol University, Bangkok, Thailand

**Keywords:** Laparoscopic cholecystectomy, Situs inversus, Gallstone disease

## Abstract

**Background:**

Situs inversus is a rare and silent autosomal recessive disorder occurring in 1:5,000 to 1:20,000 individuals. Laparoscopic cholecystectomy, a standard treatment for gallbladder disease in the general population, is very challenging in patients with situs inversus, especially for right-handed surgeons. We herein report a case involving our modified laparoscopic cholecystectomy technique for right-handed surgeons in a Thai patient with situs inversus who developed a symptomatic gallstone. We also include a short review of the literature.

**Case presentation:**

A 39-year-old female patient with dextrocardia presented with a 5-month history of episodic biliary colic. Abdominal ultrasonography revealed a left-sided gallbladder with gallstones. We performed laparoscopic cholecystectomy with our modified technique including port relocation. The operation went well, and our patient recovered satisfactorily.

**Conclusion:**

Laparoscopic cholecystectomy in patients with a left-sided gallbladder is not often confidently performed by right-handed surgeons. However, some modifications of “mirror image” ports focused on the more ergonomic port position are the keys to successful completion of this operation. The patient will thus still obtain benefits from this standard minimally invasive technique.

## Background

Situs inversus is a rare autosomal recessive condition. Its prevalence varies from 0.04% to 0.30% [1]. Situs inversus is divided into two types: situs inversus partialis, which involves the thoracic organs (dextrocardia) or abdominal viscera, and situs inversus totalis, which involves both the thoracic organs and abdominal viscera. Associated abnormalities may be found, including bronchiectasis, sinusitis, and deficient tracheobronchial cilia. This condition is referred to as Kartagener syndrome [2].

Because of the atypical “mirror image” anatomy associated with situs inversus, the gallbladder is on the left, whereas the stomach and spleen are on the right. Surgeons must be aware of this anatomy to diagnose and surgically treat patients with situs inversus who develop gallbladder disease. Various literature reviews have described occasional reports of laparoscopic management using different techniques in patients with situs inversus with symptomatic gallstones [2-4].

We reported a case of symptomatic gallstone in situs inversus Thai patient including a short review of our modified laparoscopic cholecystectomy technique for right dominant surgeon.

## Case presentation

A 39-year-old female patient with dextrocardia and a history of ventral septal defect closure presented with a 5-month history of episodic biliary colic. On physical examination, she had no fever. Her abdomen was mildly tender in the epigastrium. Liver function test results were normal. The patient underwent abdominal ultrasonography, which revealed a left-sided gallbladder with gallstones. No gallbladder wall thickening, pericholecystic fluid, or dilatation of the biliary tract was found. The patient was then scheduled for laparoscopic cholecystectomy.

**Figure 1:**
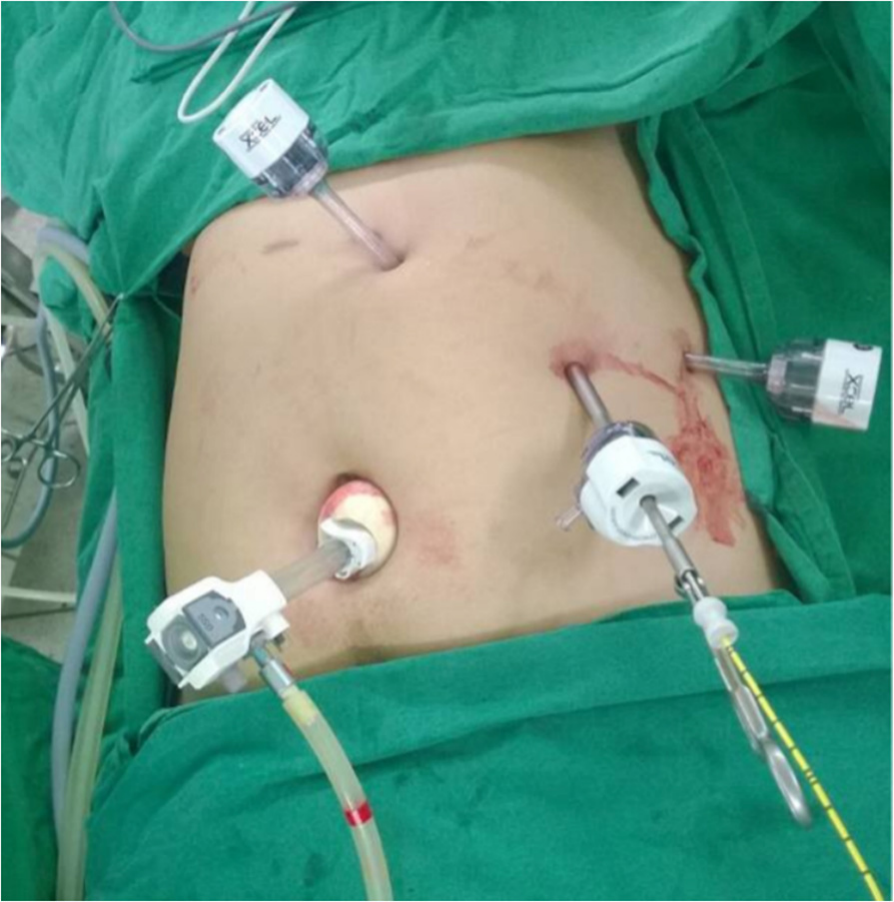
**Port positions.** 1, Camera port. 2, Epigastric port. 3, Left midclavicular port. 4, Lateral port.

In the operating room, the laparoscopic devices were equipped in the “mirror image” of the normal position for conventional laparoscopic cholecystectomy. The surgeon and the camera holder were positioned on the right side of the patient. The first assistant and the scrub nurse were positioned on the left side. We used the four-port technique (Figure 1). The first 12-mm subumbilical port was inserted, and the abdomen was insufflated with carbon dioxide to a pressure of 12 mmHg. After pneumoperitoneum had been established, a 30° laparoscope was inserted, and the presence of a left-sided gallbladder was confirmed. Three additional 5-mm ports were inserted at the epigastrium, left midclavicular line, and left anterior axillary line. The dissecting port, which is usually placed at the left midclavicular line just below the costal margin, was placed more caudally (5 cm below the costal margin).

**Figure 2:**
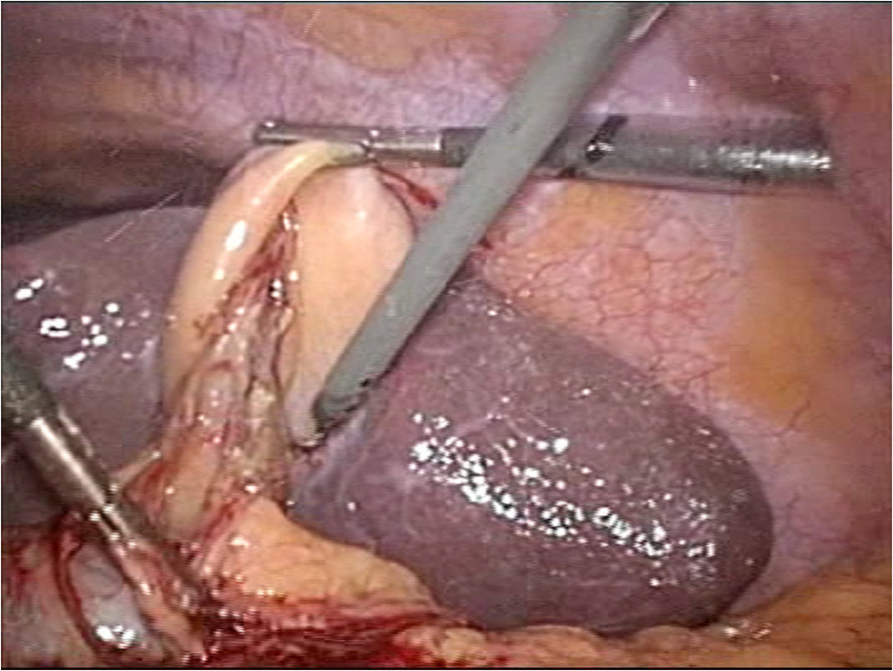
**Identification of Calot’s triangle by hook dissection in the left midclavicular port.** A, Hook dissector.

**Figure 3:**
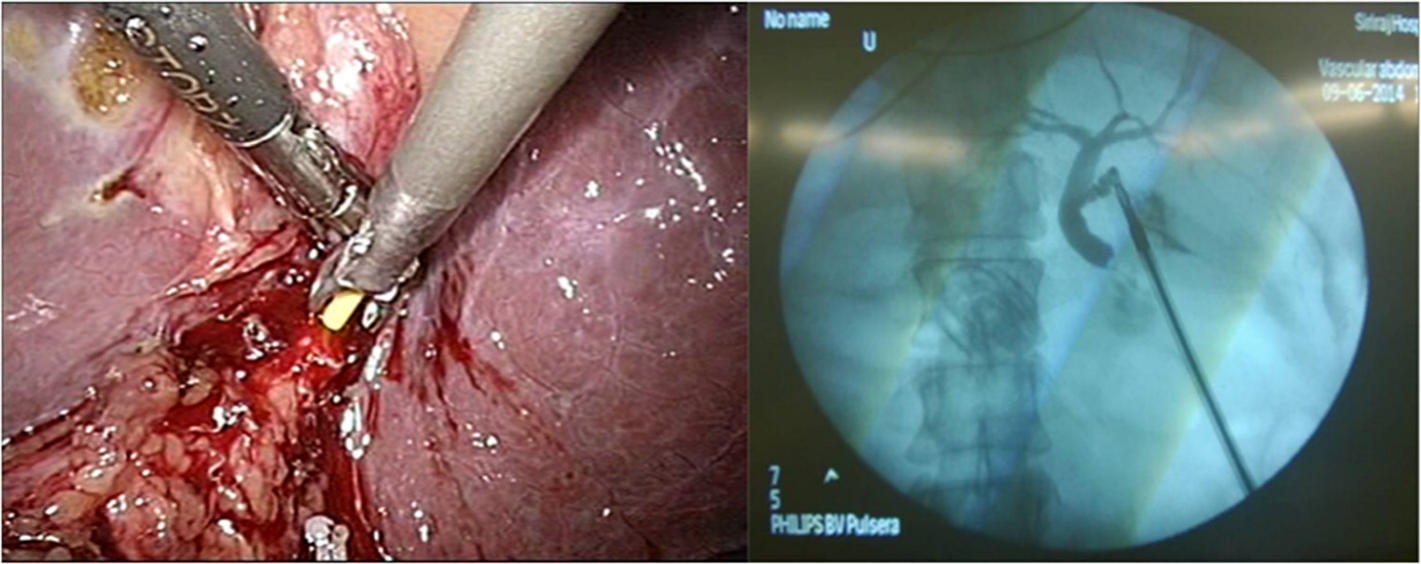
**Intraoperative cholangiography (transcystic approach) revealed a common bile duct without a filling defect.**

The gallbladder was distended and surrounded by adhesions. The first assistant grasped the fundus of the gallbladder and pulled it upward and laterally via the left lateral port. The right-handed surgeon grasped Hartmann’s pouch and pulled it laterally using his left hand via the epigastric port and dissected the adhesion using his right hand via the left midclavicular port. Identification of Calot’s triangle was difficult because the anatomy could not be clearly identified as a result of the previous inflammation (Figure 2). Therefore, intraoperative cholangiography was performed, and no abnormal variations were demonstrated (Figure 3). After the cystic duct and artery had been individually clipped and divided safely, the gallbladder was separated from its bed by electrocautery as usual and extracted in a retrieval bag through the camera port under direct vision. The operation was successfully completed in 60 minutes. The patient recovered satisfactorily and was discharged on postoperative day 2.

## Discussion

Diagnosis of gallstone disease in patients with situs inversus is difficult, especially in those with an unknown history of this condition. Because of the eccentric anatomy of the left-sided gallbladder, the clinical presentation of these patients usually involves left upper-quadrant pain; however, 30% of patients reportedly develop epigastrium pain, including our patient. Approximately 10% of patients complain of right upper-quadrant pain [5], which is a classic presentation in patients without situs inversus. Such a symptom could be troublesome in patients with previously diagnosed situs inversus. Ultrasonography is useful in these patients.

Laparoscopic cholecystectomy remains the standard operation for treatment of gallstone diseases, even in patients with situs inversus. In 1991, Campos and Sipes [6] reported the first successful laparoscopic cholecystectomy in a patient with situs inversus with a symptomatic gallstone. Several case reports and laparoscopic cholecystectomy techniques were subsequently published (Table 1). In 2008, Fernandes *et al*. [7] described a three-port technique employed by a left-handed surgeon. They placed a 12-mm subumbilical camera port, a 10-mm epigastric port, and a 5-mm left subcostal port to perform successful laparoscopic cholecystectomy. In 2010, Eisenberg [3] described a four-port technique using a “mirror image” port placement technique for conventional laparoscopic cholecystectomy. A 12-mm camera port was inserted at the umbilicus, a 5-mm port was inserted at the epigastrium, and two 5-mm ports were placed along the left subcostal line. The left-handed surgeon performed dissection through the epigastric port. There are also reports involving right-handed surgeons. Lochman *et al*. [5] and Arya *et al*. [2] performed the operation with an assistant surgeon grasping the infundibulum. The principal surgeon performed the dissection with only his dominant right hand via the epigastric port. Other reports have described the surgeon’s position on both the right side of the patient and the middle of the abducted legs of the patient (lithotomy position) to obtain a more ergonomic position [2,8].

**Table 1:** **Literature review of cases of situs inversus and laparoscopic cholecystectomy techniques**

**Ref.**	**Country**	**Number of cases**	**Diagnosis**	**Patient position**	**Number of ports**	**Dominant hand of the surgeon**	**Hand using dissection**	**Comments**	**Operative time (minutes)**
Fernandes [7]	Brazil	1	SGS	Supine	3	Left	Left hand via epigastric port	Grasping infundibulum by right hand via left mid-clavicular port	N/A
Eisenberg [3]	United states	1	SGS	Supine	4	Left	Left hand via epigastric port	Grasping infundibulum by right hand via left mid-clavicular port	85
Hall [9]	United Kingdom	1	SGS	Supine	4	Right	Right hand via left mid-clavicular port	Grasping infundibulum by left hand of the surgeon via epigastric port	65
Patle [4]	India	6	SGS and acute cholecystitis (1 case)	Lithotomy (supine in the first case)	4	Right	Right hand via left mid-clavicular port (left hand via epigastric port in the first case)	Grasping infundibulum by left hand of the surgeon via epigastric port (by right hand via left mid-clavicular port in the first case)	65
Lochman [5]	Czech Republic	1	Acute cholecystitis	Supine	4	Right	Right hand via epigastric port	The assistant grasped both fundus and infundibulum	70
Arya [2]	India	1	SGS	Supine	4	Right	Right hand via epigastric port	The assistant grasped infundibulum	95

In the conventional laparoscopic cholecystectomy technique performed by a right-handed surgeon in patients without situs inversus, the camera port was placed at the subumbilical position. Three additional ports were placed from medial to lateral as follows: an epigastric port as a dissecting port, a right midclavicular port (placed just below the costal margin) for the surgeon to grasp Hartmann’s pouch with his left hand, and a right anterior axillary port (placed just below the costal margin) for an assistant to pull the fundus of the gallbladder upward. This technique was well controlled by the surgeon when one hand of the surgeon grasped the infundibulum and the other hand performed the dissection.

In our operation, the operative equipment, surgeon’s position, and port placement were prepared as a “mirror image” to the routine laparoscopic cholecystectomy. The surgeon was positioned on the right side of the patient with situs inversus. The left midclavicular port was assigned as the dissecting port because this is more convenient to operate by the surgeon’s right hand. The surgeon’s left hand then operated the grasper through the epigastric port for the best exposure. Notably, the surgeon stands on the right side and has to move his right hand across the patient’s body to operate the dissecting port; the more cephalad the position of the port, the longer the distance the surgeon must reach, which could result in early exhaustion. Therefore, we decided to relocate the left midclavicular port 5 cm caudally, which was a more ergonomic position for the surgeon. With this modification, intraoperative precision was still preserved.

In this case, the anatomy was unclear due to the adhesion around Calot’s triangle. However, intraoperative cholangiography was still beneficial to demonstrate the ductal anatomy, as in a normal patient. The operation was successfully completed without any complications.

## Conclusions

Laparoscopic cholecystectomy in patients with a left-sided gallbladder is not often confidently performed by right-handed surgeons. However, clear identification of Calot’s triangle with or without the aid of a radiologic procedure in combination with a more ergonomic port position are the keys to successful performance of this operation. We believe that this technique will enable right-handed surgeons to be as skillful as left-handed surgeons in such cases. Moreover, patients will still obtain benefits from this standard minimally invasive technique.

## Consent

Written informed consent was obtained from the patient for publication of this case report and accompanying images. A copy of the written consent is available for review by the Editor-in-Chief of this journal.
